# Glycan composition of serum alpha-fetoprotein in patients with hepatocellular carcinoma and non-seminomatous germ cell tumour

**DOI:** 10.1038/sj.bjc.6690828

**Published:** 1999-12

**Authors:** P J Johnson, T C W Poon, N M Hjelm, C S Ho, S K W Ho, C Welby, D Stevenson, T Patel, R Parekh

**Affiliations:** Departments of Clinical OncologyChemical Pathology at the Sir YK Pao Cancer Centre, Chinese University of Hong Kong, Shatin, Hong Kong, SAR, China; Oxford GlycoSciences, Abingdon Science Park, Oxford, UK

**Keywords:** hepatocellular carcinoma, non-seminomatous germ cell tumour, alpha-fetoprotein, purification, protein structure, glycosylation

## Abstract

Although estimation of serum alpha-fetoprotein (AFP) is widely used in the diagnosis of hepatocellular carcinoma (HCC) and non-seminomatous germ cell tumours (NSGCT), the clinical usefulness of this test is limited by a low specificity. However, there exist glycoforms of AFP which may be more specific for particular tumours. Previously, detailed analysis has been prevented by the low levels of AFP in human serum. We report here the application of fluorescence labelling, sequential exoglycosidase digestion, high-performance liquid chromatography and matrix-assisted laser desorption ionization in time-of-flight mass spectrometry, to determine the glycan structures of purified serum AFP from patients with HCC and NSGCT. Eleven major glycans were found, of which seven were *N*-linked, and four were *O*-linked, to the protein backbone. The structure of the *N*-linked glycans (all of bi-antennary complex-type with varying degrees of sialylation, fucosylation and galactosylation) were consistent with those previously reported. The *O*-linked glycans (three mucin *O*-GalNAc type glycans with variable degrees of sialylation, one *O*-HexNAc monosaccharide glycan) have not previously been reported. The finding of mucin *O*-GalNAc type glycans was supported by the prediction of potential *O*-GalNAc glycosylation sites on the protein backbone by analysis of the AFP structure by molecular modelling. With knowledge of these structures it may be possible to develop more specific assays for the detection of HCC and NSGCT. © 1999 Cancer Research Campaign © 1999 Cancer Research Campaign


*British Journal of Cancer* (1999) **81**(7), 1188–1195
© 1999 Cancer Research Campaign
Article no. bjoc.1999.0828

### Glycan composition of serum alpha-fetoprotein in patients with hepatocellular carcinoma and non-seminomatous germ cell tumour

**PJ Johnson[1], TCW Poon[1], NM Hjelm[2], CS Ho[2], SKW Ho[1], C Welby[1], D Stevenson[3], T Patel[3], R Parekh[3] and RR Townsend[3]**

Departments of [1]Clinical Oncology and [2]Chemical Pathology at the Sir YK Pao Cancer Centre, Chinese University of Hong Kong, Shatin, Hong Kong, SAR, China; [3]Oxford GlycoSciences, Abingdon Science Park, Oxford, UK

**Summary** Although estimation of serum alpha-fetoprotein (AFP) is widely used in the diagnosis of hepatocellular carcinoma (HCC) and non-seminomatous germ cell tumours (NSGCT), the clinical usefulness of this test is limited by a low specificity. However, there exist glycoforms of AFP which may be more specific for particular tumours. Previously, detailed analysis has been prevented by the low levels of AFP in human serum. We report here the application of fluorescence labelling, sequential exoglycosidase digestion, high-performance liquid chromatography and matrix-assisted laser desorption ionization in time-of-flight mass spectrometry, to determine the glycan structures of purified serum AFP from patients with HCC and NSGCT. Eleven major glycans were found, of which seven were *N*-linked, and four were *O*-linked, to the protein backbone. The structure of the *N*-linked glycans (all of bi-antennary complex-type with varying degrees of sialylation, fucosylation and galactosylation) were consistent with those previously reported. The *O*-linked glycans (three mucin *O*-GalNAc type glycans with variable degrees of sialylation, one *O*-HexNAc monosaccharide glycan) have not previously been reported. The finding of mucin *O*-GalNAc type glycans was supported by the prediction of potential *O*-GalNAc glycosylation sites on the protein backbone by analysis of the AFP structure by molecular modelling. With knowledge of these structures it may be possible to develop more specific assays for the detection of HCC and NSGCT. © 1999 Cancer Research Campaign

**Keywords**: hepatocellular carcinoma; non-seminomatous germ cell tumour; alpha-fetoprotein; purification; protein structure; glycosylation

Serum alpha-fetoprotein (AFP) is a glycoprotein which has been reported to consist of 591 amino acids and one asparagine-linked bi-antennary complex-type sugar chain (Tarelli et al, 1992; Ferranti et al, 1995). The carbohydrate moiety of AFP exhibits microheterogeneity, as revealed by isoelectric focusing (Alpert et al, 1972; Burditt et al, 1994; Johnson et al, 1995) and lectin electrophoretic (Taketa et al, 1993; Shimizu et al, 1996) techniques. The sugar chain of AFP, purified from the ascitic fluid of a patient with hepatocellular carcinoma (HCC), consisted of a single *N*-linked sugar chain of the bi-antennary complex-type with a degree of heterogeneity based on differing degrees of terminal sialylation and proximal fucosylation (Figure 1) (Yoshima et al, 1980). This basic structure has been confirmed by mass spectrometric analysis of AFP derived from the hepatoblastoma cell line Hep G2 (Tarelli et al, 1992; Ferranti et al, 1995), and by paper electrophoretic and chromatographic analysis of pooled human-cord serum AFP at term pregnancy (Yamashita et al, 1993). However, the abundance of the AFP isoforms differed in the two sources of materials (Yamashita et al, 1993; Ferranti et al, 1995). Largely because of the extremely small amounts of protein available, there has been very little data on human serum AFP in patients with HCC or non-seminomatous germ cell tumours (NSGCT).

*Received 10 December 1998*
*Revised 4 May 1999*
*Accepted 13 May 1999*

*Correspondence to:* PJ Johnson

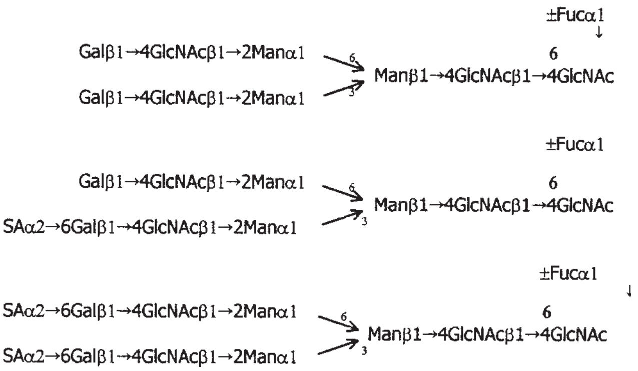

**Figure 1** Reported basic structures of the oligosaccharide of AFP

Measurement of serum AFP (reference range < 10 ng $ml^{-1}$) is used as a serological tumour marker in HCC (Johnson et al, 1978; Sheu et al, 1985; Nomura et al, 1989) and NSGCT (Lange and Fralay, 1977; Javadpour, 1980; Kay et al, 1987; Nichols et al, 1990). About 80% of patients with HCC will have levels above the reference range (Johnson et al, 1978; Sheu et al, 1985; Nomura et al, 1989). A serum concentration of greater than 500 ng $ml^{-1}$, in an HCC high incidence area and in the appropriate clinical setting, is almost diagnostic of HCC. However, modestly raised levels of AFP (10–500 ng $ml^{-1}$) may also be detected in non-malignant chronic liver diseases, thereby decreasing the specificity of the AFP test for HCC (Johnson et al, 1978; Okuda, 1986; Lok and

Lai, 1989). This poses an important clinical problem since most HCCs arise in patients with co-existing chronic liver disease (Kew and Popper, 1984; Johnson and Williams, 1987).

Several attempts have been made to identify a 'tumour-specific' glycoform, aiming thereby to improve the specificity of AFP as a diagnostic test for HCC. Previously the most successful approach in identifying tumour-specific isoforms has been based on the difference in their binding affinity to various lectins, particularly lentil lectin and concanavalin A (ConA). Indeed, differences in binding patterns of AFP of different origins were found (Smith and Kelleher, 1980; Buamah et al, 1986; Du et al, 1991). More recently, isoelectric focusing (IEF) has been used to identify directly isoforms of AFP. The AFP+II (band +II) and AFP+III (band +III) appear highly specific for HCC (Burditt et al, 1994; Ho et al, 1996) and NSGCT (Johnson et al, 1995) respectively. A unique IEF pattern is also found for the AFP which arises in patients with chronic liver disease without any evidence of malignancy (Ho et al, 1996; Johnson et al, 1997). Preliminary studies suggest that this approach may allow early, even preclinical, diagnosis of HCC in high-risk patients (Johnson et al, 1997).

In this study we have separated the sugar chains of serum AFP from patients with NSGCT and HCC by high-performance liquid chromatography (HPLC), resulting in the identification of 11 different glycans. Their compositions have been determined using sequential exoglycosidase digestion followed by chromatography or matrix-assisted laser desorption ionization in time-of-flight (MALDI-TOF) mass spectrometry. This study provides the basis for evaluating the specificity of the IEF method for separating the isoforms of AFP and for developing a routine assay for AFP with increased specificity for the detection of NSGCT and HCC.

### MATERIALS AND METHODS

Sera were collected from two patients with histologically confirmed HCC with serum AFP levels of 1 513 000 and 1 486 000 ng ml$^{-1}$, and two patients with histologically confirmed NSGCT with serum AFP levels of 14 000 and 17 000 ng ml$^{-1}$. The concentration of serum AFP was assayed by a microparticle enzyme immunoassay (MEIA, Abbott Laboratories, IL, USA). Sera were stored at –70°C until further analysis, or purification of AFP.

### Purification of AFP by one-step affinity chromatography

Rabbit anti-human AFP (Dako, Denmark) was coupled to CNBr-activated Sepharose 4B (Pharmacia Biotech, Uppsala, Sweden), overnight at 4°C in coupling buffer (0.2 M $NaHCO_3$, 0.5 M sodium chloride (NaCl), pH 8.6). Non-specific binding sites were blocked using 0.2 M glycine, pH 8.0 over 2 h at room temperature. Uncoupled antibody was removed by extensively washing the gel alternately with acetate buffer (0.1 M sodium acetate, 0.5 M NaCl, pH 4.0) and the coupling buffer. A column containing 40 ml of coupled gel was equilibrated with phosphate-buffered saline (PBS, 10 mM sodium phosphate, 100 mM NaCl, pH 6.8). The sample to be purified (approximately 400 ug AFP per 40 ml gel) was applied and run at a flow rate of 1 ml min$^{-1}$ for 30 min. The flow was then interrupted for approximately 2.5 h in order to allow for optimal binding. The column was then washed with approximately 100 ml of PBS. Bound AFP was eluted with 100 ml of 3 M NaSCN, pH 6.4. The eluate was dialysed in PBS overnight at 4°C. The resulting dialysate was concentrated to approximately 1 ml using a Centriprep 30 concentrator (Amicon, MA, USA). The 'total' AFP concentration was determined as previously described. The purity of the final AFP preparation was regarded as acceptable when only one single band, with a molecular weight of 69 kDa, was visualized on a sodium dodecyl sulphate polyacrylamide gel electrophoresis (SDS-PAGE) gel using silver staining.

### Glycan analysis of purified AFP in HCC and NSGCT

Glycans were released from an aliquot (~2 µg) of salt-free (dialysed) AFP, purified from each of the two HCC patients and two NSGCT patients, by hydrazinolysis as described previously (Patel et al, 1993). The released glycans were fluorescently labelled at the reducing terminal with 2-aminobenzamide (2-AB) by a reductive amination reaction (Bigge et al, 1995).

2-AB labelled glycan profiling was performed on a Hypersil C18 HPLC column (250 × 4.6 mm i.d.). The HPLC system consisted of a Rheodyne manual injector, Gilson 305 and 306 pumps, Gilson 805 manometric module and Gilson 811C dynamic mixer. The 2-AB labelled glycans were eluted from the column using gradient conditions by an eluent consisting of 40 mM ammonium acetate, pH 6.0 and 50 mM ammonium acetate, pH 6.0 containing 8% acetonitrile at a flow rate of 0.5 ml min$^{-1}$, as previously reported (Patel et al, 1993). Detection of 2-AB labelled glycans was achieved on a Jasco FP-920 fluorimeter (λex = 330, λem = 420 nm) and data were collected on a PE Nelson 900 Series Interface for subsequent computer-assisted analysis (Oxford GlycoSciences Data Analysis software) and simultaneously on a Kipp and Zonen chart recorder. When fractions were required, the eluate from the fluorimeter was connected to a Gilson 203 fraction collector.

To determine the structure, 2-AB labelled glycans were taken to dryness under reduced pressure (Savant) and treated with *Athrobacter ureafaciens* sialidase (100 mU in 30 µl reconstitution buffer), *Streptococcus pneumoniae* β-galactosidase (10 mU in 50 µl), Bovine testes β-galactosidase (100 mU in 30 µl), or *Streptococcus pneumoniae* β-N-acetylhexosaminidase (30 mU in 30 µl) at 37°C for 16 h. The digests were purified by passing them through a column containing 0.15 ml AG X16 triethylammonium form and 0.15 ml AG X8 acetate form and eluting with 2 ml HPLC grade water.

Gel filtration chromatography of neutral or desialyated 2-AB labelled glycans was performed on an Oxford GlycoSciences GlycoSequencer™ at a flow rate of 160 µl min$^{-1}$ in accordance with the manufacturer's instruction. A GlycoSep H HPLC column (100 × 3 mm i.d., Oxford GlycoSciences) was connected to a Gilson HPLC system as previously described and 2-AB labelled monosaccharides were eluted with gradient conditions by an eluent consisting of acetonitrile and 0.1% aqueous trifluroacetic acid containing 5% acetonitrile as previously reported (Bigge et al, 1995).

The molecular weight of 2-AB labelled glycans from AFP were determined by MALDI-TOF mass spectrometry using a PerSeptive Biosystems Voyager Elite time-of-flight mass spectrometer. Spectra were acquired with delayed extraction (100 nS) and an acceleration voltage of 20 kV. External calibration against a mixture of peptides was used. The matrix solution of alpha-cyano-4-hydroxycinnamic acid (Hewlett-Packard) was mixed with an equal volume (1 µl) of the oligosaccharide solution, vortexed and spotted onto a smooth stainless steel plate. The spectra were the average of 200 scans.

© 1999 Cancer Research Campaign 

**Table 1** The relative abundance of the 11 major glycans of AFP in NSGCT and HCC

| | Percentage of total glycan molecules | | | | | | | | | | |
|---|---|---|---|---|---|---|---|---|---|---|---|
| **Glycan** | **G1** | **G2** | **G3** | **G4** | **G5** | **G6** | **G7** | **G8** | **G9** | **G10** | **G11** |
| NSGCT case 1 | 5.3 | 11.7 | 14.6 | 9.9 | 11.3 | 5.6 | 1.5 | 9.9 | 7.7 | 11.3 | 11.3 |
| NSGCT case 2 | 8.1 | 18.1 | 12.8 | 7.1 | 13.0 | 5.0 | 2.8 | 6.1 | 4.5 | 11.1 | 11.4 |
| HCC case 1 | 4.9 | 14.6 | 11.4 | 16.8 | 19.7 | 2.4 | 2.3 | 6.2 | 5.2 | 11.5 | 5.1 |
| HCC case 2 | 2.5 | 14.9 | 7.9 | 13.2 | 13.8 | 4.7 | 9.5 | 8.0 | 8.6 | 8.0 | 9.1 |

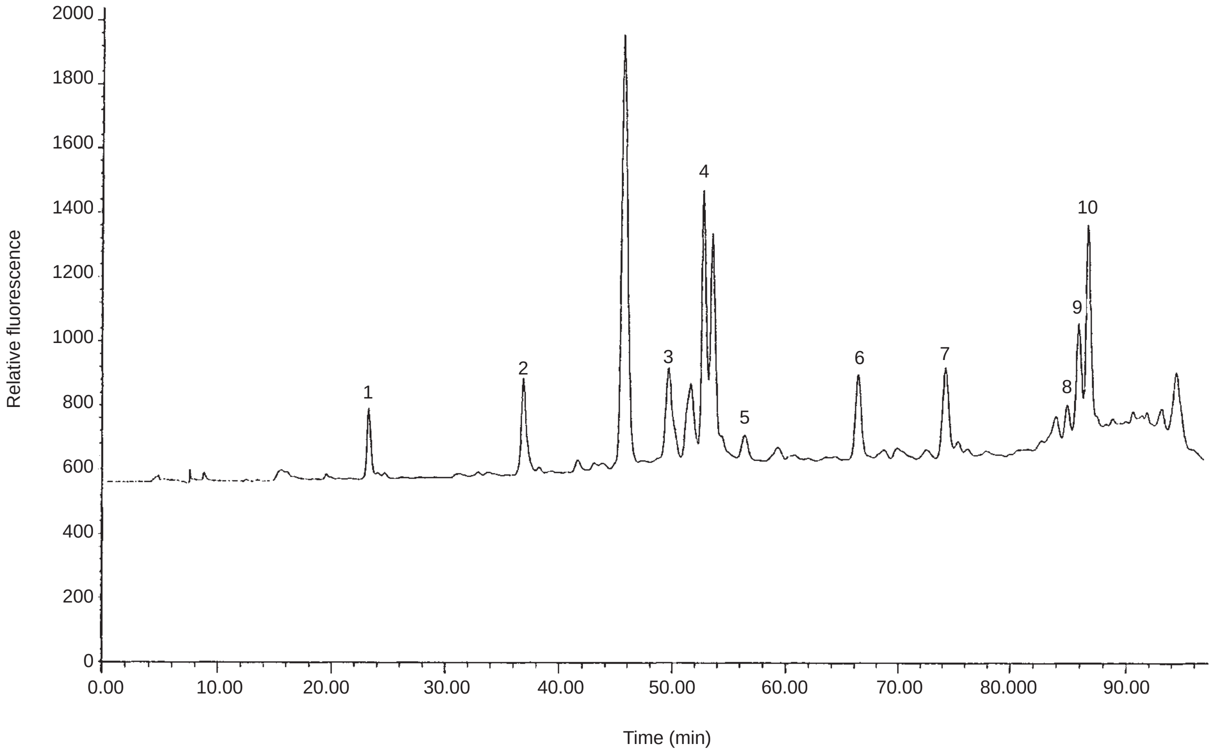

**Figure 2** The reverse phase HPLC chromatogram of 2-AB labelled glycans prepared from HCC AFP. Eleven glycans were identified in the reverse phase HPLC chromatogram of one of the HCC samples. The glycans were numbered according to the sequence of the corresponding peaks. The unlabelled peak with an elution time of 45 min was the unreacted label. Similar chromatograms were obtained for the other HCC sample, as well as the two NSCGT samples, but with different peak heights

### Localization of *O*-GalNAc glycosylation sites by computer analysis

The *O*-glycosylation sites on the AFP protein core for the linkage of *O*-GalNAc glycans were predicted by a network system, NetOglyc Version 2.0 (the Center for Biological Sequence Analysis, accessed via the Internet at http://www.cbs.dtu.dk). This system can be used to identify mucin *O*-GalNAc type *O*-glycosylation sites from a primary protein structure by comparison with data in O-GLYCBASE, a revised database of *O*-glycosylated proteins (Hansen et al, 1995, 1998; Elhammer et al, 1997). The cross-validated NetOglyc network system correctly found 83% of the glycosylated and 90% of the non-glycosylated serine and threonine residues in independent sets of tests.

### RESULTS

The 2-AB glycans were first fractionated using reverse-phase HPLC. Eleven major peak fractions were identified (Figure 2). The unlabelled peak with an elution time of 45 min was the unreacted label. Each peak fraction was pooled and the molecular size was determined by either gel filtration using the GlycoSequencer or by MALDI-TOF mass spectrometry. All glycans were desialylated prior to gel filtration and exoglycosidase sequencing. The experimentally-determined glycan size, the results of sequential exoglycosidase digestions and the elution positions of standard sialylated glycans on the reverse phase column were used to deduce the structures shown in Figure 3.

The relative abundance of the glycans in NSGT sera and HCC sera is given in Table 1. The difference in abundance of the glycans between the two groups (percentage for NSGCT glycan minus percentage for HCC) is presented in Figure 4, ranking the differences from the largest positive to the largest negative value. It is evident that the glycan profiles are similar in the sera of the two tumour types with a slight abundance of G1, G3 and G11 in NSGCT sera, and of G4, G5 and G7 in HCC sera. No attempt has been made to subject this conclusion to statistical analysis because of the small number of observations which still represent a substantial amount of analytical work.

 *© 1999 Cancer Research Campaign*

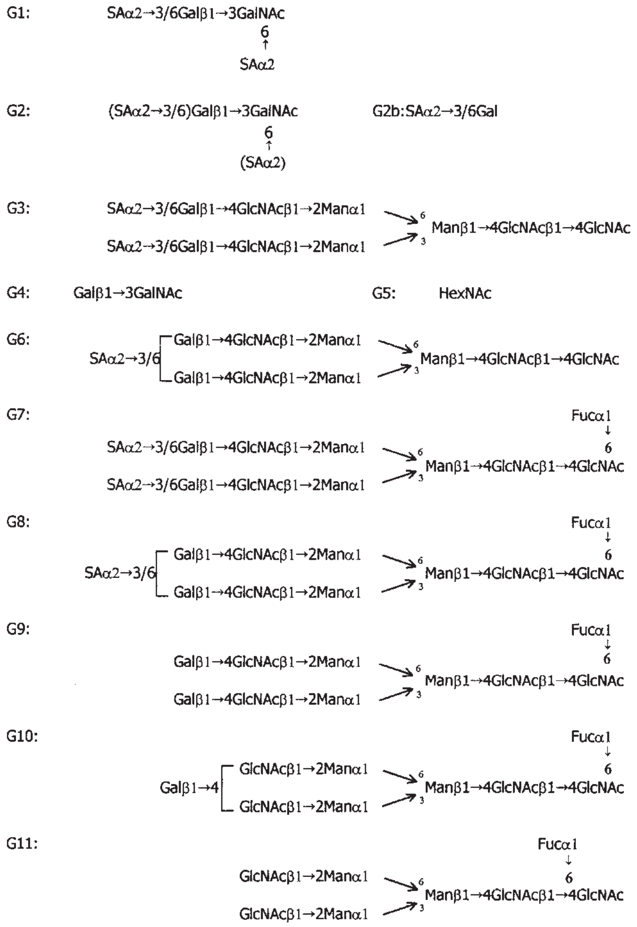

**Figure 3** The structures of the glycans found in HCC and NSGCTs

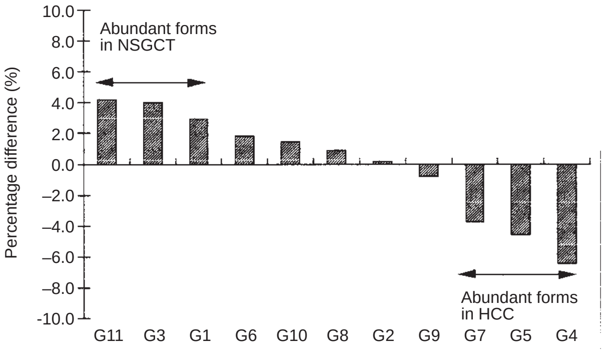

**Figure 4** Percentage difference (NSGCT minus HCC) for the relative abundance of the 11 glycans in serum AFP from patients with HCC (*n* = 2) and NSGCT (*n* = 2)

### Glycan composition

All the glycans, except G9, G10 and G11, were treated with sialidase. The neutral glycans were injected on the GlycoSequencer™ in order to obtain information on molecular size. It was apparent that G3, G6, G7, G8, G9, G10 and G11 are *N*-linked oligosaccharides having the sizes of larger than or equal to 10 glucose units (gu) whereas G1, G2 G4 and G5 are small *O*-linked oligosaccharides having a size of 1 gu or smaller after desialylation.

### Composition of *N*-linked oligosaccharides (G3, G6, G7, G8, G9, G10 and G11 glycans) – chromatography of glycan digests

Purified desialylated 2-AB labelled glycans were digested with β-galactosidase (from *Streptococcus pneumoniae*). The digests of individual glycan forms were chromatographed on the Glyco-Sequencer™. Eluted peaks from several samples were pooled and digested with β-N-acetylhexosaminidase (from *Streptococcus pneumoniae*) and re-chromatographed. Desialylated 2-AB labelled G3 and G6 glycans initially contained 11.34 gu (Figure 5) and the size was reduced to 4.8 gu after double digestion, which corresponds to a $Man_3GlcNAc_2$ pentasaccharide core, indicating that G3 and G6 are bi-antennary complex-type oligosaccharides. From the elution times of the 2-AB labelled glycans by reverse phase HPLC it could be concluded that G3 contains two sialic acid groups and G6 only one.

After the double digestion of G7, G8, G9, G10 and G11 glycans, a common core polysaccharide of 5.8 gu was identified, corresponding to a $FucMan_3GlcNAc_2$ hexasaccharide core. The degree of sialylation was derived from their elution times by reverse phase HPLC and it was found that G7 is disialylated, G8 is monosialylated and G9, G10 and G11 are not sialylated.

Further information of the structure of glycans was obtained from analysing desialylated G3 by MALDI-TOF. Mass spectra of 1783 and 1799 Da were obtained for the sodium and potassium adducts of G3, identical to their theoretical mass values and indicating their composition as $Hex_5HexNAc_4$-2-AB Na/K. The mass of sodium and potassium adducts of G10 was found to be 1767 and 1783, respectively, again identical to their theoretical mass values and indicating their composition as dHex-$Hex_4HexNAc_4$-2-AB Na/K (Figure 5). Among G9, G10 and G11, the degree of galactosylation was derived from their elution times by reverse phase HPLC. The MALDI-TOF analysis indicated that G10 is monogalactosylated. Therefore G9 is digalactosylated and G11 agalactosylated.

It can be concluded from these investigations that all the *N*-linked glycans are biantennary complex-type oligosaccharides, which differ in the degree of sialylation, fucosylation and galactosylation.

### Composition of *O*-linked oligosaccharides (G1, G2, G4 and G5 glycans) – chromatography of glycan digests

Desialylated G2 glycan could be separated into one minor and one major component by the GlycoSequencer™. The minor component was identified as D-Galactose by GlycoSepH chromatography. The major component corresponded to 1.01 gu, strongly indicating its structure as HexHexNAc-AB. The major component was further digested by β-galactosidase (from bovine testes) and fractionated by the Glycosequencer™, which resulted in one fraction representing undigested material and another fraction representing a breakdown product. The breakdown product was identified as D-*N*-acetylgalactosamine by GlycoSepH chromatography using monosaccharide standards for calibration. Therefore the major structure of the G2 glycan could be established as Galβ1→3GalNAc.

The G2 glycan was found to be monosialylated by comparing the elution times by reverse phase HPLC with that of 2-AB

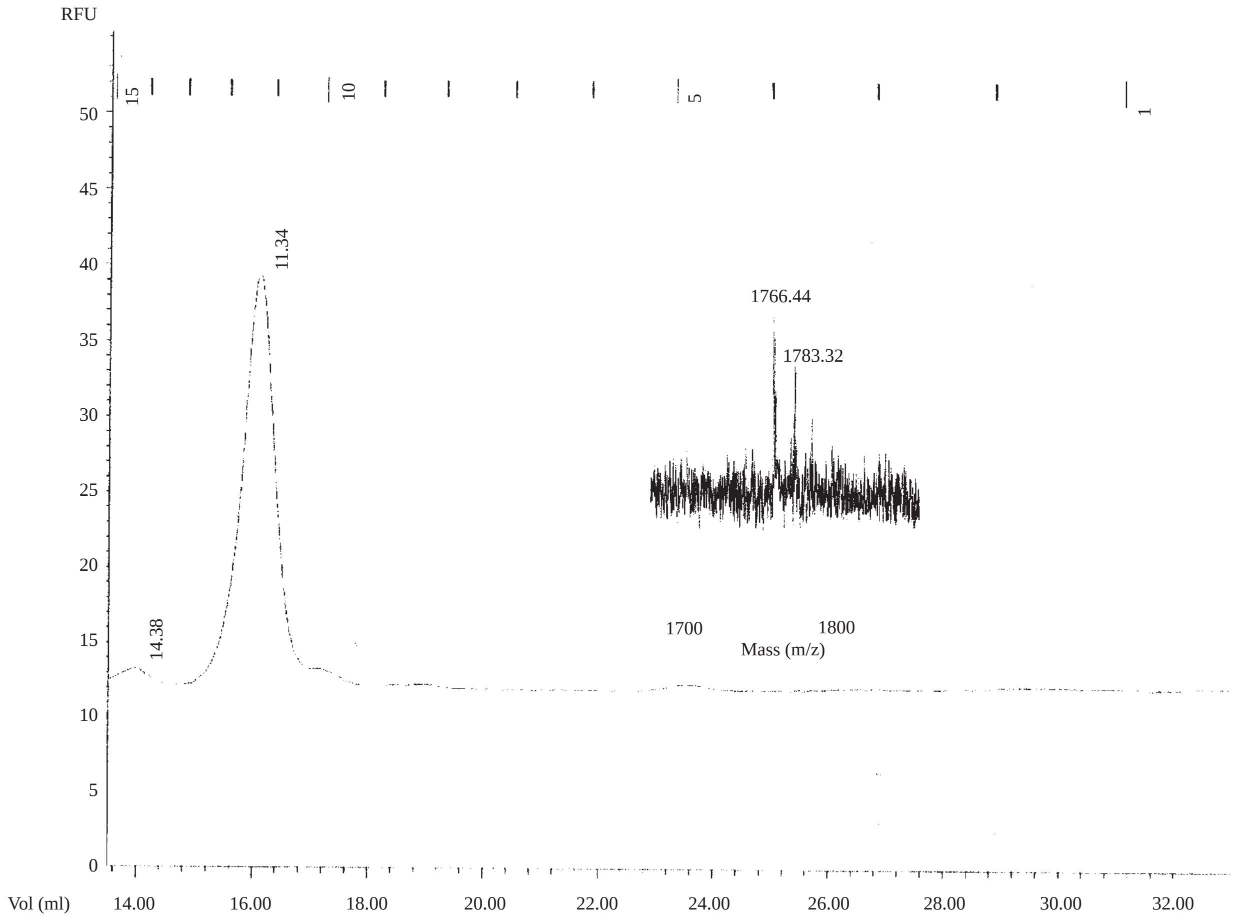

**Figure 5** Two typical examples of the chromatograms obtained in the study. GlycosequencerTM chromatogram of desialylated 2-AB labelled G3 glycan, indicating a size of 11.3 gu, and MALDI-TOF mass spectrum of GlycoSequencerTM purified 2-AB labelled G10 glycan, indicating sodium and potassium adducts with the mass of 1767 and 1783 respectively labelled 6-sialyl lactosamine. Products of G1 and G2 after sialidase digestion had the same elution time at reverse phase HPLC, which also corresponded to the elution time for G4 glycan. Therefore G1 is the disialylated form and G2 is the monosialylated form of the G4 glycan.

The non-digested G5 glycan was identified as a single monosaccharide of *N*-acetylhexosamine by GlycoSepH chromatography.

### Composition of the 11 glycans

The composition of the 11 glycans, G1–G11, is summarized in Figure 3.

### Identification of potential *O*-glycosylation sites on AFP protein core by amino acid sequence analysis

Using the NetOglyc program, which is a software for investigating protein structures for *O*-GalNAc glycosylation sites, three threonine residues in positions 132, 208 and 457 of the AFP amino acid sequence (including the signal peptide region) were identified as potential sites for *O*-GalNAc glycosylation. No serine residues were identified for the *O*-GalNAc glycosylation (Figure 6).

### DISCUSSION

Although AFP has been used as a tumour marker for the diagnosis of HCC for many years, its specificity is poor when levels fall within the ‘grey area’, i.e. 10–500 ng $ml^{-1}$. In this range differentiation between benign and malignant liver diseases cannot be made with confidence on the basis of serum AFP levels alone. There is thus a need to find a method of increasing the specificity of AFP. Recently, using IEF and immunoblotting, various AFP isoforms appearing as band +II and band +III were found to be relatively specific for HCC and NSGCT. As the pI value of a glycoprotein is greatly affected by the degree of glycosylation, it is important to study the carbohydrate composition of the glycan(s) carried by AFP before we can elucidate the molecular structure of the disease-specific isoforms.

In this study we found that the predominant AFP glycans associated with HCC and NSGCT were mixtures of 11 different *N*- and *O*-linked oligosaccharides. We would acknowledge that the number of samples was small and in order to gain sufficient material for analysis our studies were confined to subjects with very high total AFP levels. We cannot therefore be certain that our findings are necessarily generalizable to patients with lower AFP

*© 1999 Cancer Research Campaign*

```
Name:  alpha-fetoprotein     Length:  609 (including the signal peptide region)
MKWVESIFLIFLLNFTESRTLHRNEYGIASILDSYQCTAEISLADLATIFFAQFVQEATYKEVSKMVKDALTAIEKPTGD     80
EQSSGCLENQLPAFLEELCHEKEILEKYGHSDCCSQSEEGRHNCFLAHKKPTPASIPLFQVPEPVTSCEAYEEDRETFMN    160
KFIYEIARRHPFLYAPTILLWAARYDKIIPSCCKAENAVECFQTKAATVTKELRESSLLNQHACAVMKNFGTRTFQAITV    240
TKLSQKFTKVNFTEIQKLVLDVAHVHEHCCRGDVLDCLQDGEKIMSYICSQQDTLSNKITECCKLTTLERGQCIIHAEND    320
EKPEGLSPNLNRFLGDRDFNQFSSGEKNIFLASFVHEYSRRHPQLAVSVILRVAKGYQELLEKCFQTENPLECQDKGEEE    400
LQKYIQESQALAKRSCGLFQKLGEYYLQNAFLVAYTKKAPQLTSSELMAITRKMAATAATCCQLSEDKLLACGEGAADII    480
IGHLCIRHEMTPVNPGVGQCCTSSYANRRPCFSSLVVDETYVPPAFSDDKFIFHKDLCQAQGVALQTMKQEFLINLVKQK    560
PQITEEQLEAVIADFSGLLEKCCQGQEQEVCFAEEGQKLISKTRAALGV
................................................................................     80
...................................................T............................    160
...............................................T................................    240
................................................................................    320
................................................................................    400
........................................................T.......................    480
................................................................................    560
.................................................
```

Analysis of threonine residues

| Residue | No. | Potential | Threshold | Assignment |
|---|---|---|---|---|
| Thr | 16 | 0.0145 | 0.7562 | . |
| Thr | 20 | 0.0077 | 0.6935 | . |
| Thr | 38 | 0.0093 | 0.7307 | . |
| Thr | 48 | 0.0826 | 0.7798 | . |
| Thr | 59 | 0.0371 | 0.6921 | . |
| Thr | 72 | 0.0712 | 0.6781 | . |
| Thr | 78 | 0.1122 | 0.6218 | . |
| Thr | 132 | 0.8678 | 0.6566 | T |
| Thr | 146 | 0.0255 | 0.6837 | . |
| Thr | 157 | 0.0091 | 0.6891 | . |
| Thr | 177 | 0.0447 | 0.7793 | . |
| Thr | 204 | 0.2805 | 0.6999 | . |
| Thr | 208 | 0.7420 | 0.6616 | T |
| Thr | 210 | 0.0271 | 0.6545 | . |
| Thr | 232 | 0.0280 | 0.7222 | . |
| Thr | 234 | 0.0151 | 0.7153 | . |
| Thr | 239 | 0.0503 | 0.7181 | . |
| Thr | 241 | 0.5698 | 0.7095 | . |
| Thr | 248 | 0.0102 | 0.6798 | . |
| Thr | 253 | 0.0242 | 0.7267 | . |
| Thr | 294 | 0.0099 | 0.6830 | . |
| Thr | 300 | 0.0115 | 0.6839 | . |
| Thr | 306 | 0.0077 | 0.6882 | . |
| Thr | 307 | 0.0092 | 0.6994 | . |
| Thr | 387 | 0.1541 | 0.6707 | . |
| Thr | 436 | 0.0216 | 0.7006 | . |
| Thr | 443 | 0.0302 | 0.6726 | . |
| Thr | 451 | 0.3298 | 0.7225 | . |
| Thr | 457 | 0.8156 | 0.7113 | T |
| Thr | 460 | 0.0294 | 0.7072 | . |
| Thr | 491 | 0.0169 | 0.7079 | . |
| Thr | 502 | 0.0100 | 0.6838 | . |
| Thr | 520 | 0.3003 | 0.7103 | . |
| Thr | 547 | 0.0237 | 0.7267 | . |
| Thr | 564 | 0.1276 | 0.6435 | . |
| Thr | 603 | 0.0499 | 0.6440 | . |

Analysis of serine residues

| Residue | No. | Potential | Threshold | Assignment |
|---|---|---|---|---|
| Ser | 6 | 0.0003 | 0.7539 | . |
| Ser | 18 | 0.0002 | 0.6707 | . |
| Ser | 30 | 0.0108 | 0.7440 | . |
| Ser | 34 | 0.0003 | 0.6973 | . |
| Ser | 42 | 0.0017 | 0.7325 | . |
| Ser | 64 | 0.0019 | 0.6636 | . |
| Ser | 83 | 0.0042 | 0.6319 | . |
| Ser | 84 | 0.0196 | 0.6289 | . |
| Ser | 111 | 0.0027 | 0.6526 | . |
| Ser | 115 | 0.0003 | 0.6623 | . |
| Ser | 117 | 0.0009 | 0.5820 | . |
| Ser | 135 | 0.4012 | 0.7010 | . |
| Ser | 147 | 0.0053 | 0.6927 | . |
| Ser | 191 | 0.0004 | 0.6829 | . |
| Ser | 216 | 0.0007 | 0.6545 | . |
| Ser | 217 | 0.0902 | 0.6921 | . |
| Ser | 244 | 0.0038 | 0.6782 | . |
| Ser | 286 | 0.0051 | 0.7527 | . |
| Ser | 290 | 0.0118 | 0.7085 | . |
| Ser | 296 | 0.0009 | 0.6562 | . |
| Ser | 327 | 0.0017 | 0.6668 | . |
| Ser | 343 | 0.0004 | 0.6733 | . |
| Ser | 344 | 0.0002 | 0.6340 | . |
| Ser | 353 | 0.0006 | 0.7700 | . |
| Ser | 359 | 0.0210 | 0.6839 | . |
| Ser | 368 | 0.0004 | 0.7793 | . |
| Ser | 408 | 0.0003 | 0.6589 | . |
| Ser | 415 | 0.0003 | 0.6880 | . |
| Ser | 444 | 0.0100 | 0.6789 | . |
| Ser | 445 | 0.0764 | 0.6699 | . |
| Ser | 465 | 0.0006 | 0.6348 | . |
| Ser | 503 | 0.0008 | 0.6567 | . |
| Ser | 504 | 0.0012 | 0.6577 | . |
| Ser | 513 | 0.0068 | 0.6961 | . |
| Ser | 514 | 0.0020 | 0.6943 | . |
| Ser | 527 | 0.0004 | 0.6541 | . |
| Ser | 576 | 0.0003 | 0.7024 | . |
| Ser | 601 | 0.0005 | 0.6477 | . |

**Figure 6** NetOglyc 2.0 Prediction Results. The potential *O*-GalNAc glycosylation sites (T) are indicated correspondingly.

levels and we could not analyse directly the AFP arising from patients with benign liver disease. Similar problems have been faced by other workers who have attempted direct analytical studies (Yoshimaet al, 1980; Yamashita et al, 1993; Ferranti et al, 1995). However, the present study is the first to provide direct information of the glycan composition of serum AFP. Previously, it has been considered that AFP carries only a single bi-antennary complex-type *N*-glycan, varying in the degree of sialylation, galactosylation and fucosylation (Yoshima et al, 1980; Yamashita et al, 1993; Shimizu et al, 1996). We have now demonstrated that AFP can also carry *O*-linked glycans.

Some of the structures of *N*-linked glycans reported in this study have previously been identified in hepatoblastoma cell lines, cord serum and tumour ascitic fluid (Yoshima et al, 1980; Yamashita et al, 1993; Ferranti et al, 1995). Seven *N*-linked glycans found in this study were composed of the typical $GlcNAc_2Man_3GlcNAc_2$ *N*-linked heptasaccharide core, with different degrees of sialylation, fucosylation and galactosylation. The G10 (asialo monogalacto) glycan and G11 (asialo agalacto) glycan are two sugar chains of AFP not previously reported, although the presence of serum AFP isoforms with different degrees of galactosylation has been suggested indirectly by a study using sequential exoglycosidase digestion and lectin affinity electrophoresis (Shimizu et al, 1996).

Previous reports have suggested that AFP glycoforms in HCC and NSGCT carry tri-antennary or *N*-acetylglucosaminylated bi-antennary carbohydrate structures making them non-reactive to ConA (Yamashita et al, 1983; Aoyagi et al, 1991). Yamashita et al (1983) identified acetylglucosaminylated bi-antennary *N*-glycans in AFP purified from human yolk sac tumours grown in nude mice in support of this proposal. Neither tri-antennary nor acetylglucosaminylated bi-antennary *N*-glycans were found in our study. There are several possibilities to account for such differences. Firstly, the glycosylation mechanisms may have changed during the growth of the human yolk sac tumour in nude mice. Secondly, AFP with tri-antennary or acetylglucosaminylated bi-antennary *N*-glycans may not be released into the blood circulation, or may have a short half-life in the circulation. Thirdly, tri-antennary or acetylglucosaminylated bi-antennary *N*-glycans could still have been present in our samples but in amounts below our detection limit for the method used.

The identification of AFP with *O*-linked glycans is a surprising finding as several previous investigators should have detected these isoforms (Yoshima et al, 1980; Yamashita et al, 1983, 1993; Aoyagi et al, 1991). Under different experimental conditions, *O*-linked and *N*-linked glycans can be differentially released from proteins by hydrazinolysis (Patel et al, 1993). Therefore differences in our conditions (aimed to release both the *O*-linked and *N*-linked glycans) compared to those used in the previous investigations may explain the previous negative results. Three (G1, G2 and G4) of the four identified *O*-linked glycans in our study are of mucin type, composed of the typical Galβ1→3GalNAc *O*-linked disaccharide core, with different degrees of sialylation. This conclusion is independently supported by computer analysis of the primary protein structure of AFP, which predicts the presence of three potential *O*-GalNAc glycosylation sites for mucin *O*-GalNAc type *O*-glycans. For the *O*-HexNAc monosaccharide glycan (G5), characterization experiments are continuing in our laboratories.

The distribution of the different glycans linked to AFP is quite similar in sera from patients with NSGCT and HCC. There is a slightly higher abundance of the G1, G3 and G11 glycans in NSGCT AFP and of the G4, G5 and G7 glycans in HCC AFP. Although the number of observations is small, this difference is noteworthy and will be subjected to further studies. The G3 and G7 glycans are of the *N*-linked disialylated bi-antennary complex-type, but only the latter has an additional proximal fucose. A higher abundance of G7 in HCC AFP suggests that fucosylation of AFP is more pronounced in HCC. In support of this conclusion is the finding that lentil lectin-reactive AFP – a proposed diagnostic marker for HCC and not for benign liver disease – appears to be those AFP glycoforms carrying fucosylated *N*-glycans (Du et al, 1991; Taketa et al, 1993; Shimizu et al, 1996).

The G1, G4 and G5 glycans are *O*-linked, but the former is disialylated and latter two asialylated. We cannot, at the present time, explain why there should exist an abundance of the G1 glycan in NSGCT AFP, and of the G4 and G5 glycan in HCC AFP. The higher abundance of the G11 glycan in NSGCT AFP may help to explain why the AFP +III (band +III) appears as the specific isoform for NSGCTs (Johnson et al, 1995). The G11 glycan is asialylated, and the attachment of a G11 glycan (asialylated) instead of a G3 or G7 glycan (disialylated) to the AFP protein core should result in the formation of a more basic isoform.

In conclusion, the present data clearly show that besides bi-antennary complex-type *N*-glycans, there are also *O*-linked glycans on serum AFP in HCC and NSGCT. The use of the NetOglyc network system for the prediction of *O*-GalNAc glycosylation sites on AFP is a good example of using molecular modelling to support laboratory findings.

### ACKNOWLEDGEMENTS

This work was supported by a grant awarded by the Hong Kong Research Grants Council (CUHK255/96M). We are indebted to the Hong Kong Cancer Fund and the Providence Foundation Limited, Hong Kong for their continuing support of liver cancer research.

### REFERENCES

Alpert E, Drysdale JW, Isselbacher KJ and Schur PH (1972) Human α-fetoprotein: isolation, characterization, and demonstration of microheterogeneity. *J Biol Chem* **247**: 3792–3798

Aoyagi Y, Suzuki Y, Igarashi K, Saitoh A, Oguro M, Yokota T, Mori S, Nomoto M, Isemura M and Asakura H (1991) The usefulness of simultaneous determinations of glycosaminylation and fucosylation indices of alpha-fetoprotein in the differential diagnosis of neoplastic diseases of the liver. *Cancer* **67**: 2390–2394

Bigge JC, Patel TP, Bruce JA, Goulding PN, Charles SM and Parekh RB (1995) Non-selective and efficient fluorescent labelling of glycans using 2-aminobenzamide and anthranilic acid. *Anal Biochem* **230**: 229–238

Buamah PK, Harris R, James OFW and Skillen AW (1986) Lentil-lectin-reactive alpha-fetoprotein in the differential diagnosis of benign and malignant liver disease. *Clin Chem* **32**: 2083–2084

Burditt LJ, Johnson MM, Johnson PJ and Williams R (1994) Detection of hepatocellular carcinoma-specific alpha-fetoprotein by isoelectric focusing. *Cancer* **74**: 25–29

Du MQ, Hutchinson WL, Johnson PJ and Williams R (1991) Differential alpha-fetoprotein lectin binding in hepatocellular carcinoma. *Cancer* **67**: 476–480

Elhammer AP, Poorman RA and Kezdy FJ (1997) Predicting mucin-type O-glycosylation sites. In: *Techniques in Glycobiology*, Townsend RR and Hotchkiss AT (eds), pp. 271–288. Marcel Dekker: New York.

Ferranti P, Pucci P, Marino G, Fiume I, Terrana B, Ceccarini C and Malorni A (1995) Human α-fetoprotein produced from Hep G2 cell line: structure and heterogeneity of the oligosaccharide moiety. *J Mass Spect* **30**: 632–638

Hansen JE, Lund O, Engelbrecht J, Bohr H, Nielsen JO, Hansen J-ES and Brunak S (1995) Prediction of O-glycosylation of mammalian proteins: specificity patterns of UDP-GalNAc:-polypeptide N-acetylgalactosaminyl transferase. *Biochem J* **308**: 801–813

Hansen JE, Lund O, Tolstrup N, Gooley AA, Williams KL and Brunak S (1998) NetOglyc: Prediction of mucin type O-glycosylation sites based on sequence context and surface accessibility. *Glycoconjugate J* **15**: 115–130.

Ho S, Cheng P, Chan A, Leung N, Yeo W, Leung T, Lau WY, Li AKC and Johnson PJ (1996) Isoelectric focusing of alpha-fetoprotein in patients with hepatocellular carcinoma – frequency of specific banding patterns at non-diagnostic serum levels. *Br J Cancer* **73**: 985–988

Javadpour N (1980) The role of biologic tumour markers in testicular cancer. *Cancer* **45**: 1755–1761

Johnson PJ and Williams R (1987) Cirrhosis and the aetiology of hepatocellular carcinoma. *J Hepatol* **4**: 140–147

Johnson PJ, Portmann B and Williams R (1978) Alpha-fetoprotein concentration measured by radioimmunoassay in the diagnosing and excluding of hepatocellular carcinoma. *Br Med J* **2**: 661–663

Johnson PJ, Ho S, Cheng P, Chan A, Leung T and Yuen J (1995) Germ cell tumours express a specific alpha-fetoprotein variant detectable by isoelectric focusing. *Cancer* **75**: 1663–1668

Johnson PJ, Leung N, Cheng P, Welby C, Leung T, Lau WY and Ho S (1997) ‘Hepatoma-specific’ alphafetoprotein may permit preclinical diagnosis of malignant change in patients with chronic liver disease. *Br J Cancer* **75**: 236–240

Kay PH, Wells FC and Goldstraw P (1987) A multi-disciplinary approach to primary nonseminomatous germ cell tumours of the mediastinum. *Ann Thorac Surg* **44**: 578–582

Kew MC and Popper H (1984) Relationship between hepatocellular carcinoma and cirrhosis. *Sem Liver Dis* **4**: 136–146

*© 1999 Cancer Research Campaign*

Lange PH and Fralay EE (1977) Serum AFP and HCG in the treatment of patients with testicular tumours. *Urol Clin North Am* **4**: 393–406

Lok ASF and Lai CL (1989) Alpha-fetoprotein monitoring in Chinese patients with chronic hepatitis B virus infection: role in the early detection of hepatocellular carcinoma. *Hepatology* **9**: 110–115

Nichols CR, Saxman S, Williams SD, Loehrer PJ, Miller ME, Wright C and Einhorn LH (1990) Primary mediastinal nonseminomatous germ cell tumours. A modern single institution experience. *Cancer* **65**: 1641–1646

Nomura F, Ohnishi K and Tanabe Y (1989) Clinical features and prognosis of hepatocellular carcinoma with reference to serum alpha-fetoprotein levels. *Cancer* **64**: 1700–1707

Okuda K (1986) Early recognition of hepatocellular carcinoma. *Hepatology* **6**: 729–738

Patel TP, Bruce JA, Merry A, Wormald M and Parekh RB (1993) The use of hydrazine to release intact and unreduced form both N- and O-linked oligosaccharides from glycoproteins. *Biochemistry* **32**: 679–693

Sheu JC, Sung JL, Chen DS, Yang PM, Lai MY, Lee CS, Hsu HC, Chuang CN, Yang PC, Wang TH, et al (1985) Growth rate of asymptomatic hepatocellular carcinoma and its clinical implications. Gastroenterol 89: 259–266

Shimizu K, Katoh H, Yamashita F, Tanaka M, Tanikawa K, Taketa K, Satomura S and Matsuura S (1996) Comparison of carbohydrate structures for serum α-fetoprotein by sequential glycosidase digestion and lectin affinity electrophoresis. *Clin Chim Acta* **254**: 23–40

Smith CJP and Kelleher PC (1980) Alpha-fetoprotein molecular heterogeneity: physiologic correlations with normal growth, carcinogenesis and tumour growth. *Biochim Biophys Acta* **605**: 1–31

Taketa K, Endo Y, Sekiya C, Tanikawa K, Koji T, Satomura S, Matsuua S, Kawai T and Hirai H (1993) A collaborative study for the elevation of lectin-reactive alpha-fetoproteins in early detection of hepatocellular carcinoma. *Cancer Res* **53**: 5419–5423

Tarelli E, Ashcroft AE, Calam DH, Corran PH, Green BN and Wheeler SF (1992) Human alpha-fetoprotein. Molecular weight data from electrospray mass spectrometry (ESMS) analysis. *Biol Mass Spect* **23**: 315–317

Yamashita K, Hitoi A, Tsuchida Y, Nishi S and Kobata A (1983) Sugar chain of alpha-fetoprotein produced in human yolk sac tumour. *Cancer Res* **43**: 4691–4695

Yamashita K, Taketa K, Nishi S, Fukushima K and Takashi O (1993) Sugar chains of human cord serum α-fetoprotein: characteristics of N-linked sugar chains of glycoproteins produced in human liver and hepatocellular carcinomas. *Cancer Res* **53**: 2970–2975

Yoshima H, Mizuochi T, Ishii M and Kobata A (1980) Structure of the asparagine-linked sugar chains of α-fetoprotein purified from human ascites fluid. *Cancer Res* **40**: 4276–4281

© 1999 Cancer Research Campaign
